# Supplementary material for: Morbidity transition at the national and sub-national level and their determinants over the past and contemporary period in India
Source: PLoS One. 2024 Jun 21;19(6):e0304492. doi: 10.1371/journal.pone.0304492 (PMC11192379; doi:10.1371/journal.pone.0304492)
Supplement: Attachment — Submitted filename: Reviewer comments.docx [file pone.0304492.s001.docx]

Reviewer comments:

Overall, well written research with due appreciation of limitation and strengths given it’s a cross sectional nature.

Comment:

1. Among limitations it may be added that being a secondary data analysis, an inherent flaw of the representation of the age group wise and education status wise may not be exactly similar in proportional distribution. It is of a value to consider that the increasing life expectancy, urbanization and increasing access to social media via digital platforms, might have impacted, the awareness levels among the participants reporting the information, which may differ considerably, over the years from 1995 till 2018. This may, also contribute to an over estimation in the reported responses for morbidity.
